# Small RNA sequencing reveals various microRNAs involved in piperine biosynthesis in black pepper (*Piper nigrum* L.)

**DOI:** 10.1186/s12864-021-08154-4

**Published:** 2021-11-19

**Authors:** Yuanhao Ding, Yuyuan Mao, Yi Cen, Lisong Hu, Yuefeng Su, Xuemin Ma, Lu Long, Haiyan Hu, Chaoyun Hao, Jie Luo

**Affiliations:** 1 Hainan Key Laboratory for Sustainable Utilization of Tropical Bioresource, School of Tropical Crops, Hainan University, Haikou, 570228 China; 2 Spice and Beverage Research Institute, Chinese Academy of Tropical Agricultural Sciences, Wanning, 571533 Hainan China; 3 Ministry of Agriculture Key Laboratory of Genetic Resources Utilization of Spice and Beverage Crops, Wanning, 571533 Hainan China; 4 State Key Laboratory of Cotton Biology, Key Laboratory of Plant Stress Biology, School of Life Science, Henan University, Kaifeng, 475004 Henan China; 5 Hainan Provincial Key Laboratory of Genetic Improvement and Quality Regulation for Tropical Spice and Beverage Crops, Wanning, 571533 Hainan China

**Keywords:** Black pepper, miRNA, Secondary metabolism, Alkaloids, Piperine biosynthesis

## Abstract

**Background:**

Black pepper (*Piper nigrum* L.), an important and long-cultivated spice crop, is native to South India and grown in the tropics. Piperine is the main pungent and bioactive alkaloid in the berries of black pepper, but the molecular mechanism for piperine biosynthesis has not been determined. MicroRNAs (miRNAs), which are classical endogenous noncoding small RNAs, play important roles in regulating secondary metabolism in many species, but less is known regarding black pepper or piperine biosynthesis.

**Results:**

To dissect the functions of miRNAs in secondary metabolism especially in piperine biosynthesis, 110 known miRNAs, 18 novel miRNAs and 1007 individual targets were identified from different tissues of black pepper by small RNA sequencing. qRT-PCR and 5′-RLM-RACE experiments were conducted to validate the reliability of the sequencing data and predicted targets. We found 3 miRNAs along with their targets including miR166-*4CL*, miR396*-PER* and miR397-*CCR* modules that are involved in piperine biosynthesis.

**Conclusion:**

MiRNA regulation of secondary metabolism is a common phenomenon in plants. Our study revealed new miRNAs that regulate piperine biosynthesis, which are special alkaloids in the piper genus, and they might be useful for future piperine genetic improvement of black pepper.

**Supplementary Information:**

The online version contains supplementary material available at 10.1186/s12864-021-08154-4.

## Background

Black pepper (*Piper nigrum* L.) is well-known as the king of spices and was known as ‘black gold’ in ancient times due to its high commercial value and worldwide use in flavouring food. As a traditional medicinal plant, black pepper is widely used in the treatment of pain, chills, rheumatism, muscular aches, and exhaustion and to stimulate appetite [1]. As the main economic component of this plant, the spicy berries of black pepper contain extensive alkaloids, such as piperine, pellitorine, piperidine, dehydropipernolanine, piperloein B and pipernonaline. Piperine is the major pungent and principle bioactive constituent of black pepper, but the molecular mechanism and regulatory network for piperine biosynthesis have not been elucidated.

The biosynthesis of piperine originates from the condensation of piperidine with piperoyl-CoA. An enzyme called ‘piperoyl-CoA:piperidine *N*-piperoyltransferase’ catalyses the synthesis of piperine in black pepper, but the gene and protein sequences of this enzyme have not been determined. Piperidine synthesis in black pepper is from the amino acid lysine, which is first decarboxylated to cadaverine by the enzyme lysine decarboxylase (LDC) in the presence of pyridoxal phosphate (PLP). Cadaverine undergoes oxidative deamination via amine oxidase to a 5-aminopentanal, which is then cyclized to yield the *△*^1^-piperideine. Subsequently, *△*^1^-piperideine is reduced to piperidine, but the enzymes catalysing 5-aminopentanal to piperidine have not been identified. Piperoyl-CoA is derived from *p*-coumaric acid, which is generated from phenylalanine in the presence of oxygen and NADPH or tyrosine via the corresponding phenylalanine ammonia lyase (PAL). Cinnamoyl-CoA, derived from *p*-coumaric acid, is an important precursor for piperoyl-CoA biosynthesis. The piperoyl moiety is generated by the cyclization of meta-methoxy and para-hydroxyl groups on the cinnamoyl backbone. Then, a keto-ester is generated by the chain elongation of cinnamoyl-CoA with acetyl-CoA or malonyl-CoA via a Claisen-type reaction, which is then reduced by NADPH and followed by dehydration to afford piperoyl-CoA [2–4].

MiRNAs are a class of endogenous small noncoding RNAs that are known as regulators of gene expression at the posttranscriptional level and function in the biosynthesis of secondary metabolites in plants [5]. In *Arabidopsis*, miR156-targeted *SPL9* negatively regulates anthocyanin accumulation by repressing the expression of *flavonoid 3′-hydroxylase* (*F3′H*), *dihydroflavonol reductase* (*DFR*), and other anthocyanin biosynthetic genes through a MYB-bHLH-WD40 transcriptional activation complex [6]. Recently, miR828 and miR858 targeting *MYB114* were found to promote anthocyanin biosynthesis in high anthocyanin grape lines [7]. Moreover, numerous miRNAs, such as miR172i, miR5298b, miR396b and miR828a, were found to be involved in phenylpropanoid biosynthesis in *Podophyllum hexandrum* [8], *Taxus* [9] and *Diospyros kaki* Thunb [10]. For terpenoid biosynthesis, miR156-targeted *SPL9* can positively regulate the expression of *terpene synthase 21* (*TPS21*) by directly binding its promoter, thereby controlling the synthesis of sesquiterpenoids [11]. As nitrogen-containing low-molecular-weight compounds, alkaloids are derived from amino acids and are highly diverse and heterogeneous in nature. It has been reported that some miRNAs participate in alkaloid biosynthesis, such as benzylisoquinoline in opium poppy and nicotine in tobacco [12, 13].

Till the date, only a few miRNA studies have investigated the *Piper* genus [14–17]. Most of these studies have been focused on aspects, such as miRNA structure, family belonging and conservatism, but there is little known about their functions in regulating developmental processes and in controlling metabolism in black pepper. In this study, high-quality small RNA sequencing was performed to identify the temporal and spatial expression of miRNAs and their regulation in controlling metabolism in black pepper. A total of 24 samples were prepared, including 3 biological replicates of 8 different tissues of root, stem, leaf, flower and 4 stages of fruits. The results showed that nearly two-thirds of known miRNAs are specifically up- or downregulated in fruits. Functional analyses have suggested that some miRNAs and their corresponding targets might be involved in black pepper resistance to biotic stress and piperine biosynthesis. Our findings indicated the potential functions of miRNAs in black pepper in regulating the biosynthesis of various secondary metabolites and provide new insights into miRNA functions in black pepper.

## Results

### High-throughput small RNA sequencing of *P. nigrum*

**Fig. 1:**
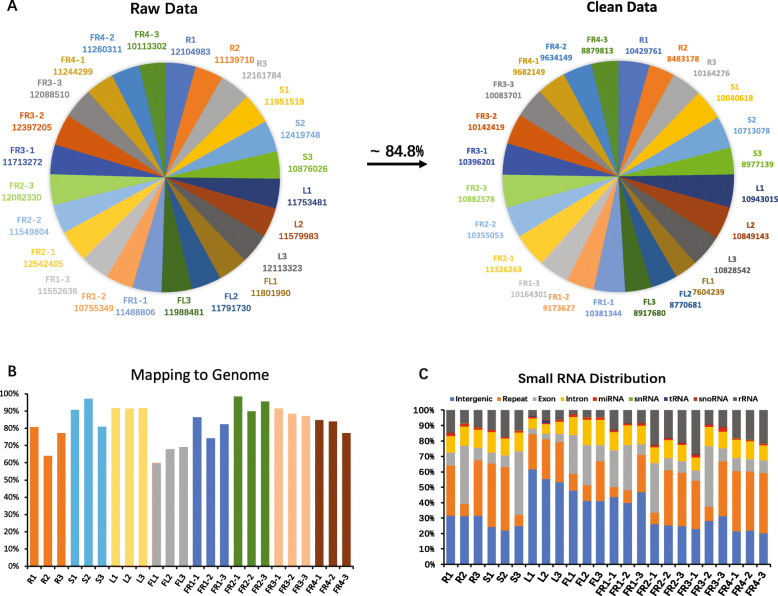
Small RNA sequencing data generated by Illumina HiSeq 2500 from 24 *P. nigrum* samples. **A.** Raw and clean data produced by sequencing. An average of 84.8% clean data were obtained. **B.** Percentage of clean data mapped to the genome of *P. nigrum*. Most samples could reach 80% mapping ratios except for the root and flower. The Y axis indicates the percentage, and the X axis indicates the samples. **C.** Annotation and distribution of mapped tags. miRNAs accounted for nearly 1.14% of all mapped tags. Data represent the means of 3 biological replicates. R: Root; S: Stem; L: Leaf; FL: Flower; FR1–4: fruits from 2, 4, 6, 8 months, respectively

**Fig. 2:**
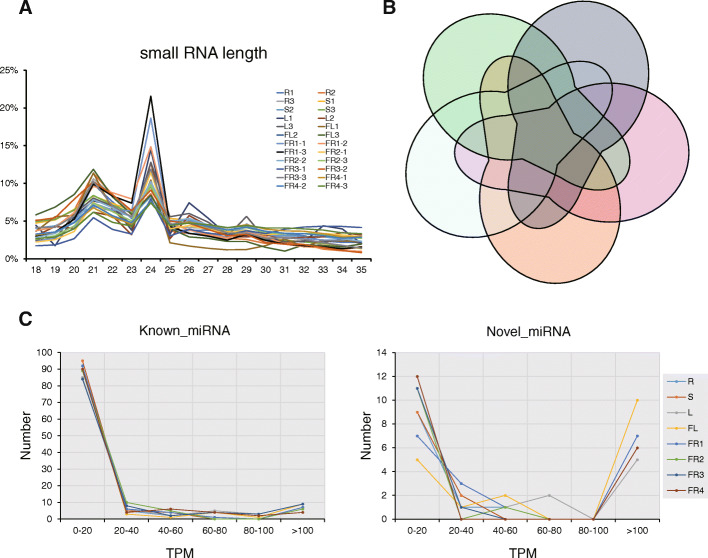
Small RNA length distribution, specifically expressed miRNA distribution and expression level ranges of known and novel miRNAs in *P. nigrum***. A.** Small RNA length in all samples from 18 to 35 nt. **B.** Specific expressed miRNAs distributed in different tissues. miRNAs from 4 stages of fruits (FR1–4) were merged together to FR represented miRNAs expressed in the fruit. **C.** Number of identified known and novel miRNAs in *P. nigrum*. X-axis represents the ranges of TPM values, y-axis represent the numbers of miRNAs

To identify miRNAs potentially involved in secondary metabolism especially in alkaloid metabolism in *P. nigrum*, we constructed 24 small RNA libraries including the roots (R), stems (S), leaves (L), flowers (FL) and 4 stages of fruits (FR1–4, fruits after flowering for 2, 4, 6 and 8 months) from *P. nigrum* cultivar Reyin1 with three biological replicates. Nearly 11.6 million raw reads and 9.9 million clean reads (~ 84.8%) were finally obtained (Fig. 1A). In this study, 80% of clean tags were mapped to the *P. nigrum* genome, which was then used for known and novel miRNA identification, and small RNA annotation (Fig. 1B). The results showed that reads from intergenic regions, repeats, exons, introns and rRNA together occupied most (~ 86%) of the mapped reads, and 1.14% of the mapped reads were annotated as miRNAs (Fig. 1C). Small RNA lengths of clean reads ranging from 18 to 35 nt were counted and are shown in Fig. 2A. The highest and second abundances were at 24 and 21 nt lengths in all samples, indicating a normal distribution of small RNA lengths compared to other small RNA sequencing studies [18, 19].

### Known and novel miRNAs identification in *P. nigrum*

To illustrate miRNAs involved in secondary metabolism, known and novel miRNAs were identified using clean reads mapped to the *P. nigrum* genome (methods described in the ‘Bioinformatics analysis’ section). In total, 128miRNAs, including 110 known and 18 novel miRNAs, were detected from small RNA sequencing data (Table 1, Supplementary Table 1). In each sample, nearly 90 known and 14 novel expressed miRNAs could be detected (Table 1). A Venn diagram was made to show the expression of miRNAs in each sample (miRNAs from 4 samples of fruit were merged together, Fig. 2B). Most miRNAs (91/128) were found to be commonly expressed in all samples, and tissue-specific expressed miRNAs occupied a minor part (Fig. 2B). The expression levels of miRNAs were then calculated as transcripts per kilobase million (TPM). The numbers of known and novel miRNAs in each sample were counted according to TPM values ranging from 0 to > 100 and are shown in Fig. 2C. Among the known miRNAs, miRNAs with low expression (TPM < 20) occupied the most part (> 80 in 110), and only ~ 10 miRNAs were highly expressed (TPM > 100). Among the novel miRNAs, the amount of low- and highly expressed miRNAs was similar in all samples (Fig. 2C). These results reveal that highly expressed miRNAs are considered occupied the minor part in *P. nigrum*, which is also reflected in Supplementary Table 1 showing TPM values.

**Table 1:** Summary of known and novel miRNAs as detected by small RNA sequencing of *P. nigrum*

	#number (All)		#number (TPM > 5)	
	Known miRNA	Novel miRNA	Known miRNA	Novel miRNA
R	87	14	34	13
S	89	13	30	13
L	89	13	41	13
FL	90	17	42	15
FR-1	98	16	39	14
FR-2	92	14	38	14
FR-3	92	14	43	14
FR-4	92	13	29	13
Total	110	18	68	16

**Fig. 3:**
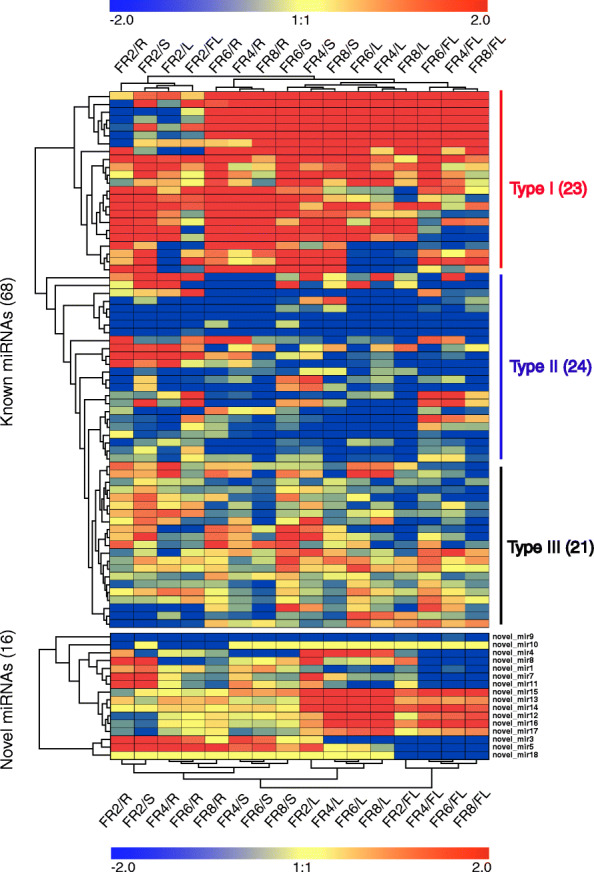
Heatmaps of known and novel miRNAs. Heatmap showing fold changes of 68 known miRNAs and 16 novel miRNAs between tissues. For known miRNAs, 3 miRNA clusters (type I-III) were classified, including nearly 1/3 upregulated (23/68, type I) and 1/3 downregulated (24/68, type II) DEMs in fruits and 1/3 DEMs (21, type III) without obvious expression trends in tissues. Heatmap showing fold changes of 16 novel miRNAs between tissues. Log_2_(fold change ratios) of fruits to other tissues were used. Red indicates upregulation, blue means downregulation and yellow indicates no change. The heatmap was made by Genesis

To further classify the miRNAs, the miRNAs with low expression with TPM < 5 in all samples were first removed, leaving 68 known and 16 novel differentially expressed miRNAs (DEMs, Table 1). The fruit of black pepper is rich in notable secondary metabolites, especially alkaloids, compared with other tissues. To understand the miRNA expression patterns between fruits and other tissues (R, S, L, FL), a heatmap of known and novel miRNAs (Fig. 3) was made using Genesis [20] (http://genome.tugraz.at/) using the log_2_(fold changes) values (Supplementary Table 2). The results showed that DEMs between fruits and other tissues were clustered together, indicating special expression patterns between samples. All 68 known miRNAs were divided into 3 clusters (Type I-III) including 23 up- (Type I) and 24 down- (Type II) regulated DEMs in fruits, which together occupied nearly 2/3 of the DEMs in the known miRNAs (Fig. 3). The remaining DEMs (Type III, 21) did not show obvious expression trends between tissues. For 16 novel miRNAs, only novel_miR9 was found downregulated in the 4 stages of fruit. Novel_miR10 and novel_miR18 were found specially expressed in root and flower, respectively (Fig. 3). Furthermore, 6 novel miRNAs (novel_miR12 - miR17) were found highly expressed in leaves and flowers. The remaining novel miRNAs were downregulated or upregulated in 1 or 2 tissues.

### Expression validation of the miRNA expression profiles via qRT-PCR

**Fig. 4:**
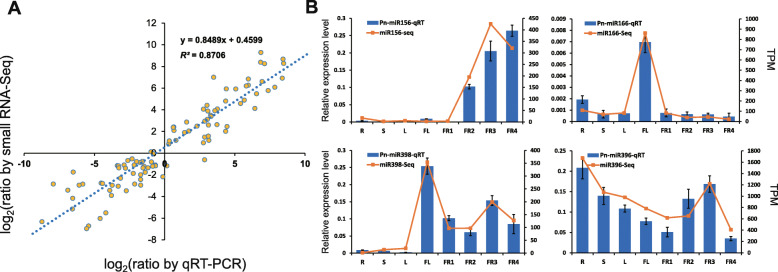
qRT-PCR validation of the miRNA expression levels detected by small RNA sequencing. **A.** Correlations of miRNA expression profiles between small RNA sequencing and qRT-PCR. Log_2_(ratios) of qRT-PCR and small RNA-Seq were used to draw the scatter diagram. The trend line, regression equation and correlation coefficient were generated automatically in EXCEL. **B.** qRT-PCR analysis of miR156, miR166, miR398 and miR396 in *P. nigrum*. *PnHis3* was used as an internal control. Data are the means ± SD from 3 biological replicates

A total of 15 miRNAs (including 10 known and 5 novel miRNAs) with high or low expression levels were selected randomly to perform qRT-PCR to validate the reliability of the small RNA sequencing. Log_2_(ratios) of qRT-PCR and small RNA-Seq were used to show the correlation between each other. Overall, qRT-PCR measurements were generally in agreement with the small RNA sequencing (*R*^2^ = 0.8706; Fig. 4A). The qRT-PCR results of 4 relatively highly expressed miRNAs derived by small RNA sequencing are shown in Fig. 4B. We found that the expression levels of four miRNAs were relatively high according to small RNA sequencing. The transcript of miR156 increased gradually from R to FR; the expression of miR166 was extremely high in FL; miR398 was preferentially expressed in FL and the fruits; and miR396 showed a relatively high expression level in all tissues but decreased gradually from R to FR1. Additionally, the expression level of 4 novel miRNAs were also detected and showed in Fig. S1. We found that novel miR5, novel miR7 and novel miR18 were both highly expression in FL, and novel miR9 highly expressed in R, S and L, which was largely corresponding to the trends of TPM values (Fig. S1).

### MiRNA target prediction; GO and KEGG pathway analysis

**Fig. 5:**
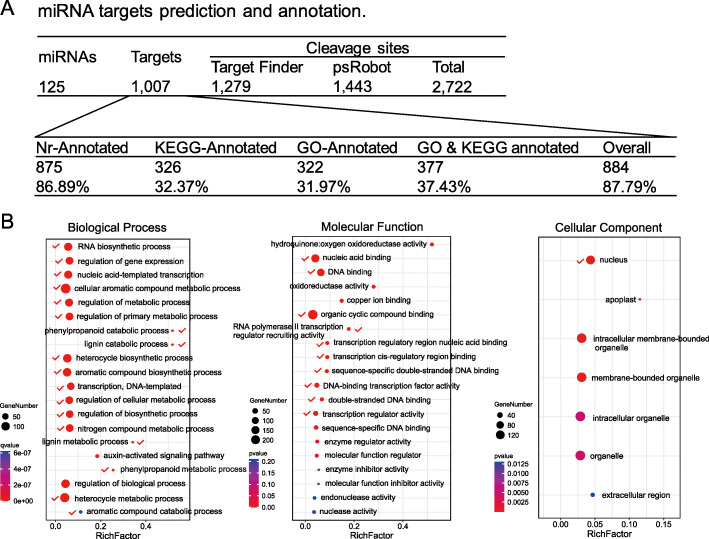
Gene Ontology (GO) and KEGG annotation of predicted targets by TargetFinder and psRobot. **A.** MiRNA target prediction and annotation by TargetFinder and psRobot. **B.** GO enrichment of targets. Targets related to metabolic or transcriptomic processes are highlighted by red ticks. Ball size represents target numbers. The colours from red to blue or green represent *P*-values ranging from low to high

The function of miRNAs depends on the posttranscriptional regulation of their targets; therefore, target prediction and validation are the basis for learning the function of miRNAs. A total of 1007 individual targets (including 2722 cleavage sites) of 125 miRNAs were predicted by TargetFinder and psRobot (Fig. 5A, Supplementary Tables 3, 4). In total, 87.79% of targets were annotated, including GO [21] (~ 31.97%) and KEGG [22] (~ 32.37%) annotations. All these annotated targets were then used for Blast2GO and KEGG pathway enrichment analysis to classify their functions (Supplementary Table 5 and 6). We were interested in the miRNAs regulating the targets that are related to secondary metabolism; thus, correlatively enriched metabolic pathways were considered (Fig. 5B). Our results showed that many metabolic processes were significantly enriched (highlighted by red ticks) such as phenylpropanoid metabolism, lignin metabolism, and cyclic compound biosynthetic processes. Additionally, the transcriptional processes were highly enriched, such as the regulation of gene expression, nucleic acid-templated transcription, and transcription processes (Fig. 5B), which implied that miRNAs and their targets participated in various metabolic processes. Furthermore, targets of miRNAs on molecular functions were clearly enriched in DNA and nucleic acid binding activity, and the most enriched cellular component was the nucleus (Fig. 5B), indicating that enriched targets may function in regulating gene transcription and expression. Taken together, our results suggest that these predicted targets of our miRNAs are likely to regulate metabolic processes and gene expression.

According to KEGG analysis, the most enriched pathways were plant hormone signal transduction, transcription factors and plant-pathogen interactions (Supplementary Fig. 2), which implied that many miRNAs and their targets participated in the response to biotic stress in black pepper. The candidate targets of miRNAs include transcription factors (TFs), such as MYB and ARF, and some important proteins in disease resistance and hormone signal transduction, such as RPS2, PRM1, BRI1, TIR1 and DELLA (Supplementary Table 7). For black pepper living in tropical environmental conditions, enriched plant-pathogen interactions and plant hormone signal transduction pathways are reasonable. Furthermore, all 1007 targets were then used for TF prediction using PlantTFDB [23] (http://planttfdb.cbi.pku.edu.cn/). There were 5 highly abundant TF families, MYB (34), SBP (19), ARF (18), GRF (15) and NAC (11) (Supplementary Table 8). These TFs are mostly involved in the processes of plant growth and abiotic or biotic stress responses, indicating that many miRNAs and their targets might participate in the response to biotic stress resistance in black pepper. In addition, 111 targets occupying nearly 22.6% of the KEGG annotated targets (326) of 74 miRNAs were found to be involved in various metabolic pathways (Supplementary Table 7). The miRNAs and their corresponding targets related to the following pathways are listed in Table 2: phenylpropanoid biosynthesis; tropane, piperidine and pyridine alkaloid biosynthesis; isoquinoline alkaloid biosynthesis; anthocyanin biosynthesis; and phenylalanine, tyrosine and tryptophan biosynthesis. Genes such as *4CL* and *CUAO* were found to participate in various metabolic processes. In addition, the homologous genes of 4 *PERs* (*Pn24.105*, *Pn7.365*, *Pn16.989* and *Pn4.2519*) involved in catalysing the synthesis of lignin monomers were predicted as the targets of miR396 (Supplementary Fig. 3A), the binding site located at a relatively conserved region of *PER* that encodes the amino acid sequences of E/QCPGVVS (Supplementary Fig. 3B). Furthermore, 2 miRNAs target *CUAO* and *TYDC* participate in alkaloid metabolism (tropane, piperidine and pyridine alkaloid biosynthesis and isoquinoline alkaloid biosynthesis). One of the 4 miRNAs, miR169, regulated the 4 homologous genes of *UGT79B1* (*Pn21.922*, *Pn21.931*, *Pn21.925* and *Pn21.928*), which are involved in anthocyanin biosynthesis.

**Table 2:** MiRNAs and corresponding targets involved in 5 secondary metabolic pathways of interest

Pathway	miRNAs	Annotations	Target ID
Phenylpropanoid biosynthesis	miR166	4CL	Pn9.356
	miR396	PER	Pn24.105; Pn7.365; Pn16.989; Pn4.2519
	miR397	CCR	Pn15.2487
Tropane, piperidine and pyridine alkaloid biosynthesis	novel_mir15	CUAO	Pn2.2577, Pn2.2494
Isoquinoline alkaloid biosynthesis	miR159	TYDC	Pn6.2746
	novel_mir15	CUAO	Pn2.2577, Pn2.2494
Anthocyanin biosynthesis	miR169	UGT79B1/A3G2XYLT	Pn21.922; Pn21.931; Pn21.925; Pn21.928
Phenylalanine, tyrosine and tryptophan biosynthesis	miR5021	CM	Pn8.1140
	novel_mir11	AroB/DHQS	Pn16.732; Pn24.508
	novel_mir18	TRPE	Pn1.2305
Phenylalanine metabolism	miR166	4CL	Pn9.356
	novel_mir15	CUAO	Pn2.2577, Pn2.2494
	miR408	AMIE	Pn37.138; Pn3.1189

### Expression analysis and 5′ RLM-RACE validation of candidate miRNAs and their targets related to piperine biosynthesis

**Fig. 6:**
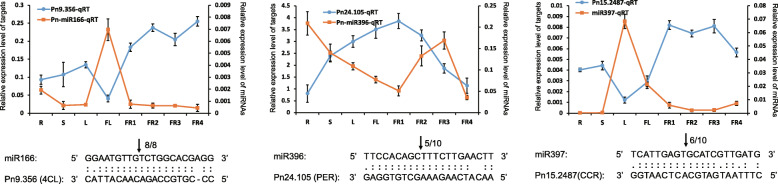
qRT-PCR and 5′ RLM-RACE validations of miRNAs and targets related to piperine biosynthesis. Arrows indicate the cleavage sites for miRNA with the number above suggesting the ratio of corrected cleavage events (out of 10) validated by 5′ RLM-RACE. *PnHis3* was used as an internal control

According to published studies, two precursors for piperine biosynthesis, piperoyl-CoA and piperidine were generated from phenylamine and lysine metabolism, respectively [24]. In this study, a total of 8 targets (*4CL* (*Pn9.356*) was regulated by miR166; 4 *PERs* (*Pn24.105*, *Pn7.365*, *Pn16.989* and *Pn4.2519*) were regulated by miR396; *CCR* (*Pn15.2487*) was regulated by miR397; *CUAO* (*Pn2.2577*, *Pn2.2494*) were regulated by novel_mir15), which were found to directly participate in phenylamine and lysine metabolism, implying that these targets might be involved in the biosynthesis of piperine. To validate the expression relationships between these miRNAs and targets and to characterize the predicted cleavage sites, qRT-PCR and 5′ RLM-RACE were performed. Finally, 3 targets (*4CL*, *PER*, *CCR*) were found to have relatively opposite expression trends with their miRNAs (Fig. 6). The cleavage sites validated by 5′ RLM-RACE generally agreed with the predicted results (Fig. 6). In addition, we found that *4CL* and *CCR* were both highly expressed in fruits compared with other tissues, and the corresponding miRNAs showed a relatively lower expression trend, which indicated that the metabolic processes from phenylalanine to caffeyl alcohol, coniferyl alcohol, 5-OH coniferyl alcohol and sinapyl alcohol were enhanced. On the other hand, the expression level of *PER* was low in the fruits, implying that the biosynthesis of lignin might be suppressed. Thus, these findings suggest that piperine biosynthesis was enhanced in the fruits under the regulation of miRNAs.

### Various miRNAs and targets participate in regulating piperine biosynthesis

**Fig. 7:**
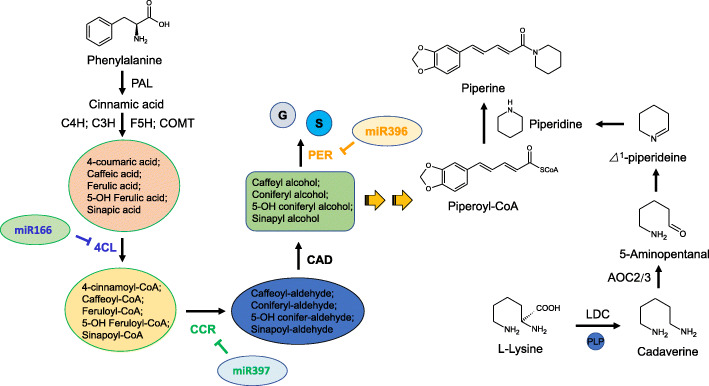
Schematic showing miRNAs and targets involved in piperine biosynthesis. G: G lignin monomers; S: S lignin monomers. Chemical structures were drawn using Chemsketch 2.0

The simplified schematic in Fig. 7 shows the regulation of miRNAs and their targets participating in piperine biosynthesis in black pepper. Piperine is generated from piperoyl-CoA and *△*^1^-piperideine [24]. The enzyme catalysing piperine synthesis is known as ‘piperoyl-CoA:piperidine *N*-piperoyltransferase’. Piperoyl-CoA is derived from the phenylalanine metabolism pathway, but the direct precursors still unclear. In addition, lignin monomers also come from phenylalanine, and we suspect that the biosynthesis of piperine and lignin share the same origin to some degree. The fruits of black pepper have a highly lignified seed coat, which reminds us of the potential possible relationships under lignin and piperine biosynthesis. In this study, we found that 3 miRNAs (miR166, miR397, and miR396) can regulate *4CL*, *CCR* and *PER*, thereby participating in the regulation of piperoyl-CoA biosynthesis. Furthermore, the enzyme that initiates the synthesis of piperidine originating from lysine and the enzymes that catalyse the last two steps from 5-aminopentanal to piperidine have not been identified.

## Discussion

Piperine is the main pungent component present in the spice berries of black pepper, and it has been confirmed to have various bioactivities, such as immunomodulatory, anti-asthmatic, stimulatory, anticancer and antimicrobial properties [25–27]. MiRNAs regulating targets involved in secondary metabolism have been widely found in many plant species in recent years [7, 12, 13, 28], but few studies have investigated black pepper. In this study, we identified miRNAs related to plant development and secondary metabolism via small RNA sequencing in 8 tissues from black pepper, which provides new insights into miRNA functions in regulating secondary metabolites and alkaloid biosynthesis in plants.

### MiRNAs participating in alkaloids biosynthesis

Alkaloids are low-molecular-weight nitrogenous organic compounds that are mostly derived from amino acids and are highly diverse and heterogeneous in nature. Alkaloids have a variety of biological activities and are used as pharmaceuticals, stimulants, narcotics, and poisons. MiRNAs regulating the biosynthesis of alkaloids have been reported in some species in recent years. In opium poppy, three miRNAs, pso-miR13, pso-miR2161 and pso-miR408, have been found to be involved in benzylisoquinoline alkaloid biosynthesis by regulating three functional enzymes, 7-O-methyltransferase (7-OMT), *S*-adenosyl-L-methionine:3′-hydroxyN-methylcoclaurine 4′-O-methyltransferase 2 (4-OMT) and reticuline oxidase-like protein (in charge of the conversion of S-reticuline to (S)-scoulerine in benzylisoquinoline alkaloid biosynthesis), respectively [12]. In tobacco, 4 unique tobacco-specific miRNAs have been predicted to target key genes of the nicotine biosynthesis and catabolism pathways; nta-miRX17 targets quinolinate phosphoribosyltransferase 1 (*QPT1*); nta-miRX27 targets *QPT2*; nta-miRX20 targets *CYP82E4*; and nta-miRX19 targets *NAC_148* [13]. Moreover, the expression of nta-miRX27 was found to be inhibited by an endogenous target mimic (eTM), nta-eTMX27, resulting in the upregulation of *QPT2,* thereby enhancing nicotine biosynthesis in topping-treated tobacco plants. In our study, 3 miRNAs and their targets were found to be involved in the biosynthesis of piperine (Fig. 7); miR166 targets *4CL*; miR396 targets *PER*; and miR397 targets *CCR*. In previous studies, *4CL* was predicted to be regulated by miR156, miR1858 and miR396 [29, 30], although without any experimental evidence. On the other hand, the biosynthesis of piperine was derived from phenylalanine, which is also the precursor for many secondary metabolites, such as lignans, anthocyanins, and flavonoids. In our findings, the upregulated miR396 in fruits resulted in a relative downregulation of *PER*, which mediated the biosynthesis of lignin monomers (Fig. 6). At the same time, the expression of *4CL* and *CCR* was enhanced, while their corresponding miRNAs showed downregulation in fruits (Fig. 6). Our findings indicate that numerous miRNAs, as well as their functional enzyme targets, participate in balancing the biosynthesis of lignin and piperine in the fruits of black pepper. In addition, several other studies on different species have reported the miRNAs involved in alkaloid biosynthesis [8, 31, 32]. In conclusion, miRNAs always function as regulators in balancing plant growth, stress resistance and metabolism processes including the synthesis of various alkaloids in different species.

### MiRNAs modulate secondary metabolism in plants

In previous studies, many miRNAs have been found to function directly or indirectly in regulating secondary metabolism in plants. In *Arabidopsis*, miR156 has been found to target *SPLs*, which indirectly regulates the accumulation of anthocyanins by preventing the expression of anthocyanin biosynthetic genes through destabilization of a MYB-bHLH-WD40 transcriptional activation complex [6]. Interestingly, miR156-targeted SPL can also progressively regulate sesquiterpene biosynthesis in *Arabidopsis* by directly binding the promoter of *TPS21* (sesquiterpene synthase gene) and activating its expression [11], which indicates that the same miRNA and its target can take part in regulating multiple metabolic processes. In addition, miR828 and miR858 have also been found to be involved in regulating anthocyanin and flavonol accumulation by target VvMYB114 [7]. In our study, miR169 participated in anthocyanin biosynthesis in black pepper by target UGT79B1 (Table 2), an anthocyanidin 3-O-glucoside 2″’-O-xylosyltransferase catalysing pelargonidin 3-O-glucoside to pelargonidin 3-O-beta-D-sambubioside. In our study, 74 miRNAs were identified as targeting 111 genes involved in metabolic pathways (Supplementary Table 7), indicating the powerful functions of miRNAs in regulating the metabolic pathway in black pepper. With the development of high-throughput sequencing technology, an increasing number of miRNAs have been found to participate in regulating various primary or secondary metabolic processes in plants. For example, the overexpression of miR8154 and miR5298b in *Taxus* cells resulted in the upregulation of key genes in Taxol, phenylpropane, and flavonoid biosynthesis [9]. In persimmon, miR395p-3p can target bHLH mRNA, and in turn, regulate the structural genes that influence proanthocyanidin accumulation [10]. In *H. capsica*, miR6194 can target F3H, which is a key enzyme in the biosynthesis of flavonols, anthocyanidins and proanthocyanidins [33]. In addition, many miRNAs have been identified that are involved in lignin synthesis, sugar and acid metabolism, and hormone signalling during pear fruit development [29]. These findings suggested that miRNA is an important regulator for controlling metabolic processes at the posttranscriptional level.

## Conclusions

As a common phenomenon in plants, miRNA regulation of secondary metabolism enhances the ability of plants to respond to the environment. Our work has proven that various miRNAs involved in the biosynthesis of many secondary metabolites in black pepper, especially in regulating piperine, a special alkaloid present in plants within the Piper genus, which provides different perspectives and abundant information on the regulation of plant metabolism and development by miRNAs.

## Methods

### Plant materials and total RNA extraction

Eight tissues (root, stem, leaf, flower, and the fruits from 2, 4, 6, and 8 months after pollination) were collected from 13 year-old black pepper cultivars (*Piper nigrum* L. cv. ‘Reyin No.1’) from the Spice and Beverage Research Institute of Chinese Academy of Tropical Agricultural Science, Wanning, Hainan, China. All samples were harvested in the field at 10:00 a.m., frozen in liquid nitrogen immediately and then sent to Novogene (Beijing, China) for RNA extraction and library construction. A total of 24 samples with 3 biological replicates were prepared.

Total RNA was prepared using the miRcute Plant miRNA Isolation Kit DP504 (Tiangen, Beijing, China) according to the manufacturer’s instructions. The extracted total RNA was then monitored by an Agilent Bioanalyzer 2100 system (Agilent Technologies, CA, USA), 1% agarose gels and a NanoPhotometer® spectrophotometer (IMPLEN, CA, USA) for integrity, purity and concentration examination to guarantee high-quality total RNA for small RNA sequencing.

### Small RNA library construction

Approximately 3 μg of total RNA from each sample was prepared for small RNA library construction. In brief, the total RNA was first purified by polyacrylamide gel electrophoresis (PAGE) to obtain small RNAs that were 18–30 nt in length. Then, the NEB 3′ SR adaptor was ligated to the 3′ end of the small RNA fragments; double-stranded DNA adaptor was transformed; 5′ end adapters were then ligated to the 5′ ends of the small RNA fragments; and cDNA was generated by a reverse transcription reaction. Several rounds of PCR amplification were performed, and the products were purified by agarose gel to obtain enough fragments from 100 to 120 bp for Illumina sequencing. The quality of purified products was assessed using the Agilent Bioanalyzer 2100 system.

### Bioinformatics analysis

The raw reads of small RNA sequencing were first filtered to obtain clean data for later analysis via the following steps: removing low quality reads, reads containing poly-N, reads shorter than 18 nt, reads with 5′ adapter contaminants and reads without a 3′ adapter insert. Then, all clean reads were aligned to the *P. nigrum* genome [34] by Bowtie2 [35]. Mapped small RNA tags were first used for secondary structure prediction and then aligned to the miRBase21.0 [36] database (http://www.mirbase.org/) for known miRNA identification. At the same time, all clean reads were aligned to the GenBank [37] (ftp://ftp.ncbi.nlm.nih.gov/genbank/) and Rfam 12.1 [38] (http://rfam.janelia.org/) databases to annotate other small RNAs, including rRNA, tRNA, small nuclear RNA (snRNA) and small nucleolar RNA (snoRNA). Reads mapped to the genome without any annotations were then used for novel miRNA prediction by mirDeep2 [39] and PIPmiR1.1 [40]. Subsequently, psRobot [41] and TargetFinder [42] were used for computational prediction of the miRNA targets. The expression level for all identified miRNAs was calculated by transcripts per million (TPM) using the following formula: mapped read count/total reads × 1,000,000. To evaluate the expression changes between samples, 3 biological replicates were first merged by the mean values of TPM (mTPM) and then calculated by log_2_(mTPM of sample 1/mTPM of sample 2). A heatmap was generated by Genesis software (http://genome.tugraz.at/) using the fold change values.

### Gene ontology and KEGG pathway enrichment

To explore the functional category distribution and pathway enrichment of the miRNA targets, Gene Ontology (GO) [43] term analysis (www.geneontology.org) and KEGG [22] analysis were performed using Blast2GO [21] software and KOBAS 3.0 [44] (http://kobas.cbi.pku.edu.cn/). All GO categories and KEGG pathways were screened under the condition of a *P*-value < 0.05.

### 5′ RLM-RACE used for target cleavage site identification

To verify the predicted target cleavage sites, RNA ligase-mediated rapid amplification of the cDNA ends (RLM-RACE) was performed using the FirstChoice® RLM-RACE Kit AM1700 (Invitrogen, Thermo Fisher Scientific, Massachusetts, USA). Briefly, the protocol from the manufacturer’s instructions involved following steps: (1) total RNA (5 μg) from all samples (24 samples containing biological repeats) were equally mixed together; (2) RNA mixtures were treated with tobacco acid pyrophosphatase (TAP) to remove the 5′ cap from the mRNA; (3) a 5′ RACE adapter was ligated to decapped mRNA without calf intestinal phosphatase (CIP) treatment; (4) reverse transcription was performed by GeneRacer Oligo dT primers to produce cDNA; (5) PCR amplification was performed with adapter primers and 3′ gene-specific primers (designed from 3′ UTR); and (6) PCR products were then ligated to pGEM T-easy vector (Promega, Madison, WI, USA) for later sequencing by TsingKe (Beijing, China). Primers were designed by Primer 5.0 and synthesized by Tianyi Huiyuan, Wuhan, China.

### Quantitative real-time reverse transcriptase-polymerase chain reaction (qRT-PCR)

To quantify the expression levels of miRNAs and target genes, stem-loop RT-PCR was performed according to reported methods [45, 46]. The reverse transcription reaction was performed as follows: (1) 2 μg of total RNA was first mixed with the primers (1 μl Oligo dT primers and 1 μl stem-loop primers), 1 μl of dNTP and appropriate RNA-free water up to 13 μl; (2) the mixture was incubated at 65 °C for 5 min to align primers to the RNA, and it was transferred to ice immediately for 2 min; (3) 4 μl of 5× FS buffer, 1 μl RNaseOUT (40 units/μl), 1 μl of DTT and 1 μl of SuperScript III RT (200 units/μl) were added to the mixture in a 20-μl reaction solution; (4) reverse transcription reactions were performed via the following steps: 16 °C for 30 min, 60 cycles of 30 °C for 30 s, 42 °C for 30 s, 50 °C for 1 s, and then 85 °C for 5 min to stop the reactions. For the qRT-PCR experiments, cDNA products were first diluted into 50× solutions. Subsequently, 10 μl of diluted cDNA was combined with 10 μl of FastStart Essential DNA Green Master (Roche, Basel, Switzerland) qRT-PCR master mix containing 0.25 μM forward and reverse primers. qRT-PCR was performed in a QuantStudio 5 system (Thermo Fisher Scientific, Massachusetts, USA) with 40 cycles of 95 °C for 5 s and 60 °C for 30 s. *PnHis3* was used as the internal control. The primers used in this study are listed in Supplementary Table 9.

## Supplementary Information

**Additional file 1.**

**Additional file 2.**

**Additional file 3.**

**Additional file 4.**

**Additional file 5.**

**Additional file 6.**

**Additional file 7.**

**Additional file 8.**

**Additional file 9.**

**Additional file 10.**

**Additional file 11.**

**Additional file 12.**

## Data Availability

The small RNA sequencing data used in this study can be found in the National Center for Biotechnology Information (NCBI) SRA database under accession number: PRJNA589468.

## Abbreviations

4CL: 4-coumarate--CoA ligase
5′-RLM-RACE: 5′ RNA ligase-mediated rapid amplification of cDNA ends
AMIE: Amidase
AroB/DHQS: 3-dehydroquinate synthase
CCR: Cinnamoyl-CoA reductase
CIP: Calf intestinal phosphatase
CM: Chorismate mutase
CUAO: Copper amine oxidase
DFR: Dihydroflavonol reductase
DHQ-SDH: 3-dehydroquinate dehydratase / shikimate dehydrogenase
F3′H: Flavonoid 3′-hydroxylase
GO: Gene Ontology
KEGG: Kyoto Encyclopedia of Genes and Genomes
LDC: Lysine decarboxylase
miRNA: microRNA
NADPH: Nicotinamide adenine dinucleotide phosphate
PAL: Phenylalanine ammonia lyase
PER: Peroxidase
PLP: Pyridoxal phosphate
qRT-PCR: Quantitative real-time reverse transcriptase-polymerase chain reaction
snRNA: Small nuclear RNA
snoRNA: Small nucleolar RNA
TAP: Tobacco acid pyrophosphatase
TPM: Transcripts Per Kilobase Million
TPS21: Terpene synthases 21
TRPE: Anthranilate synthase component I
TYDC: Tyrosine decarboxylase
UGT79B1/A3G2XYLT: Anthocyanidin 3-O-glucoside 2″‘-O-xylosyltransferase
