# Supplementary material for: Small RNA sequencing reveals various microRNAs involved in piperine biosynthesis in black pepper (Piper nigrum L.)
Source: BMC Genomics. 2021 Nov 19;22:838. doi: 10.1186/s12864-021-08154-4 (PMC8603596; doi:10.1186/s12864-021-08154-4)
Supplement: Supplementary file 1 — Additional file 1. [file 12864_2021_8154_MOESM1_ESM.pdf]

Additional file 1: Supplementary Figure 1

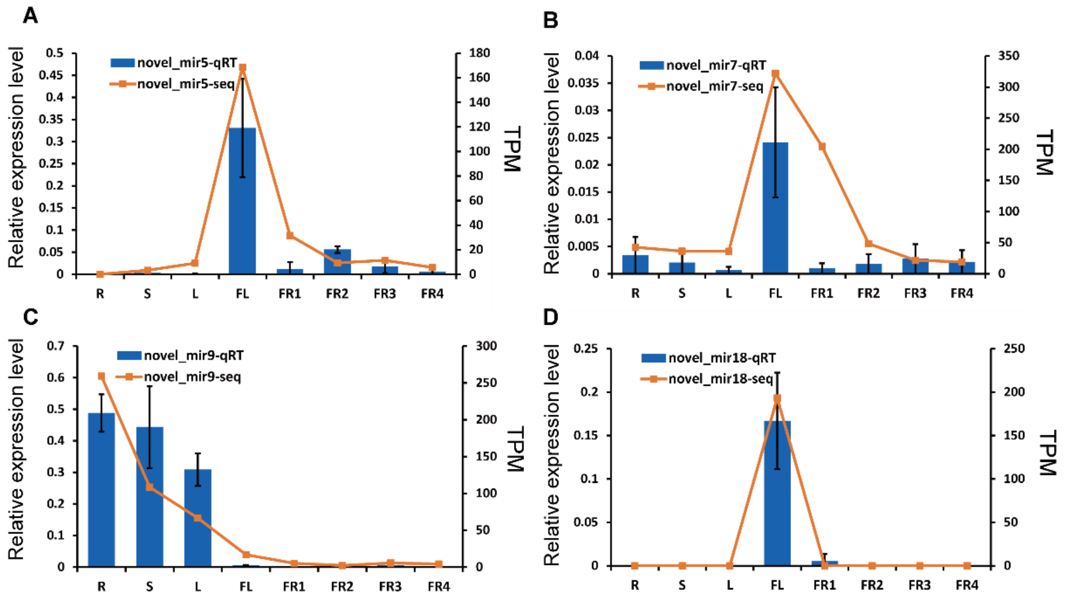

Supplementary Figure 1: qRT-PCR analysis of novel miR5, novel miR7, novel miR9 and novel miR18 in *P. nigrum*. *PnHis3* was used as an internal control. Data are the means ± SD from 3 biological replicates.
