# Supplementary material for: Small RNA sequencing reveals various microRNAs involved in piperine biosynthesis in black pepper (Piper nigrum L.)
Source: BMC Genomics. 2021 Nov 19;22:838. doi: 10.1186/s12864-021-08154-4 (PMC8603596; doi:10.1186/s12864-021-08154-4)
Supplement: Supplementary file 2 — Additional file 2. [file 12864_2021_8154_MOESM2_ESM.pdf]

Additional file 2: Supplementary Figure 2

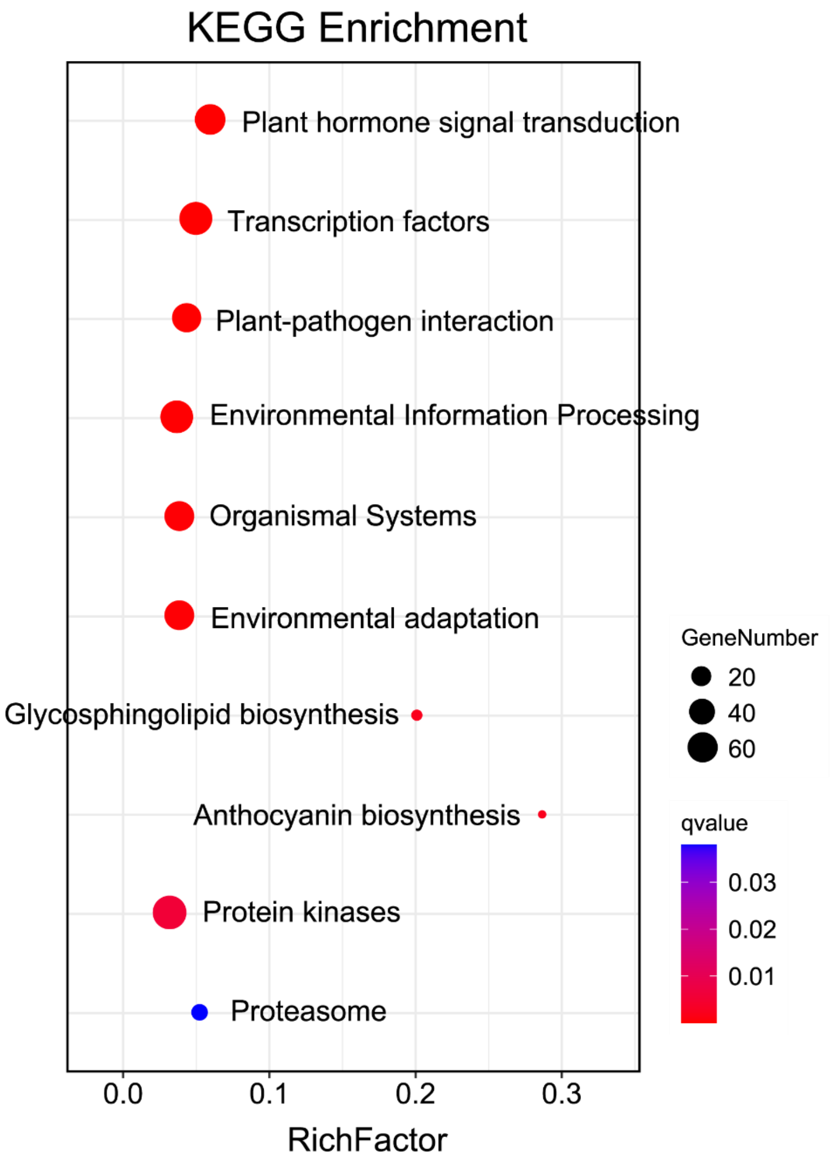

Supplementary Figure 2: KEGG pathway enrichment of targets. Enriched KEGG pathways with P-values < 0.05 were used for map construction. Ball size represents target numbers. The color from red to green represents P-values ranging from low to high.
