# Supplementary material for: Small RNA sequencing reveals various microRNAs involved in piperine biosynthesis in black pepper (Piper nigrum L.)
Source: BMC Genomics. 2021 Nov 19;22:838. doi: 10.1186/s12864-021-08154-4 (PMC8603596; doi:10.1186/s12864-021-08154-4)
Supplement: Supplementary file 3 — Additional file 3. [file 12864_2021_8154_MOESM3_ESM.pdf]

Additional file 3: Supplementary Figure 3

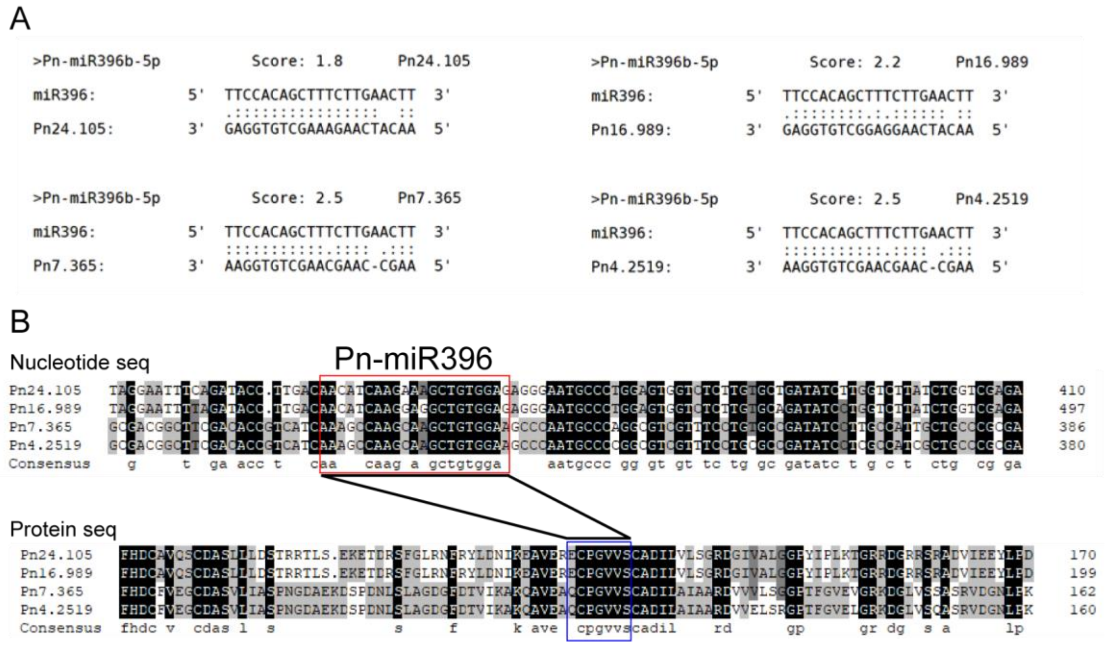

Supplementary Figure 3: Cleavage sites, nucleotide and protein sequences of PER regulated by miR396. A. The predicted cleavage sites of PER regulated by miR396. **B.** Nucleotide and protein sequences of PER targets binding with miR396.
